# Norm, gender, and bribe-giving: Insights from a behavioral game

**DOI:** 10.1371/journal.pone.0189995

**Published:** 2017-12-22

**Authors:** Tian Lan, Ying-yi Hong

**Affiliations:** 1 School of Psychology, Beijing Normal University, Beijing, China; 2 Department of Marketing, The Chinese University of Hong Kong, Hong Kong, China; Middlesex University, UNITED KINGDOM

## Abstract

Previous research has suggested that bribery is more normative in some countries than in others. To understand the underlying process, this paper examines the effects of social norm and gender on bribe-giving behavior. We argue that social norms provide information for strategic planning and impression management, and thus would impact participants’ bribe amount. Besides, males are more agentic and focus more on impression management than females. We predicted that males would defy the norm in order to win when the amount of their bribe was kept private, but would conform to the norm when it was made public. To test this hypothesis, we conducted two studies using a competitive game. In each game, we asked three participants to compete in five rounds of creative tasks, and the winner was determined by a referee’s subjective judgment of the participants’ performance on the tasks. Participants were allowed to give bribes to the referee. Bribe-giving norms were manipulated in two domains: norm level (high vs. low) and norm context (private vs. public), in order to investigate the influence of informational and affiliational needs. Studies 1 and 2 consistently showed that individuals conformed to the norm level of bribe-giving while maintaining a relative advantage for economic benefit. Study 2 found that males gave larger bribes in the private context than in the public, whereas females gave smaller bribes in both contexts. We used a latent growth curve model (LGCM) to depict the development of bribe-giving behaviors during five rounds of competition. The results showed that gender, creative performance, and norm level all influence the trajectory of bribe-giving behavior.

## Introduction

To solve the problem of corruption, it is crucial to understand the psychological mechanism of bribery [1]. Many companies report having to pay bribes to win business deals—from 15–20% in some developed countries up to 40% in some developing countries (e.g., China, Russia, and Mexico) [2]. Nevertheless, there is a lack of behavioral studies on bribery. The few extant studies on the topic are based on anonymous interviews [3], ratings of perceptions of bribery behaviors, or bribery scenarios [4]. Very few studies have assessed actual bribery behaviors directly. This may be because, like other legally liable behaviors, individuals are often reluctant to display or report these behaviors openly. In addition, there are ethical concerns for researchers to evoke and/or assess legally liable behaviors as well. To fill this knowledge gap, the present research examined bribery in an incentivized game designed to simulate a bribery situation. The main goal was to examine how situational and personal factors may influence bribe-giving behavior. Specifically, we examined how gender and social norms (e.g., the amount of bribes others give) affected individuals’ bribe-giving behavior within the behavioral game. Because much research has argued that bribery is a cultural phenomenon [5] [6] [7], it is important to understand how social norms, an important underpinning of culture [8], affect bribe-giving. Previous literature has shown inconclusive results when it comes to gender differences in corruption and bribe-giving, so we set out to examine the effect of gender in the present study as well. To begin, we’ll review the pertaining literature on social norms and gender differences.

### Social norms

The economic perspective views bribe-giving as an instrumental, rational action [9] based on economic utility and profit maximization; that is, bribe-giving functions as a means to achieve optimal economic gains for an individual or organization, often at the expense of others [10] [11] [12]. Political economists have proposed that, beyond immediate economic benefits, bribery can also contribute to the building of social networks (e.g., donation to the campaign funds of a candidate during election), which in turn will bring about long-term benefits for individuals and organizations [13]. Further, Weisel & Shalvi [14] found that cooperation with a partner on equal terms can actually promote corruption. This sort of corruption may contribute to large-scale social ills [15].

Other studies have taken the cultural perspective and argued that bribery and corruption are the results of shared socio-cultural norms, and thus bribery is more prevalent in some (e.g., collectivist) cultures than in other (e.g., individualist) cultures [16] [17]. Other research has argued that a “culture of corruption” is created through individuals’ internalization of certain culturally shared values, beliefs, or practices [18]. For example, cultural values such as reciprocity [5] [19] and responsibility diffusion [17] have been shown to increase individuals’ intention to bribe and their actual bribe-giving behavior [6] [20].

Interestingly, studies have examined correlational links between corruption at the country level and honesty at the individual level; that is, whether people from a severely corrupted country would also be likely to display dishonest behaviors. Results from these studies, however, are mixed. On the one hand, it was found that people who reside in countries that show severe rule violation (including corruption, tax evasion, political fraud) indeed display high individual dishonest behavior in laboratory [7] and natural [6] experiments. On the other hand, in another study, participants from countries that have high corruption level displayed similarly low frequency of dishonest behavior as those from countries that have low corruption level [21]. Here, the contexts in which the dishonest behavior was displayed (whether face-to-face communication is involved or not) matter more than which country the respondents were from.

In short, previous research has focused mainly on the economic and cultural influences of bribe-giving behavior. In the present research, we sought to bridge these two approaches using theories of social influence. Research from social psychology has provided abundant evidence that people are often influenced by other’s actions [22] [23]. For example, in a classic experiment, Asch [22] showed that participants conform to others’ judgment of the length of a line despite that judgment’s obvious incorrectness. Similarly, d’Adda and colleges [24] also showed that subjects follow other players’ fairness beliefs in economic experiments. By the same token, social norms—how most others respond in a certain situation—can provide both *informational* influences and *affiliational* influences [25] [26] [27] [28]. Specifically, social norms surrounding bribery provide information about its social appropriateness and potential for punishment [29] [30]. In addition, the normative level of bribe (the average amount of other people’s bribes) serves as useful information for participants deciding on their own bribe payment.

**Fig 1:**
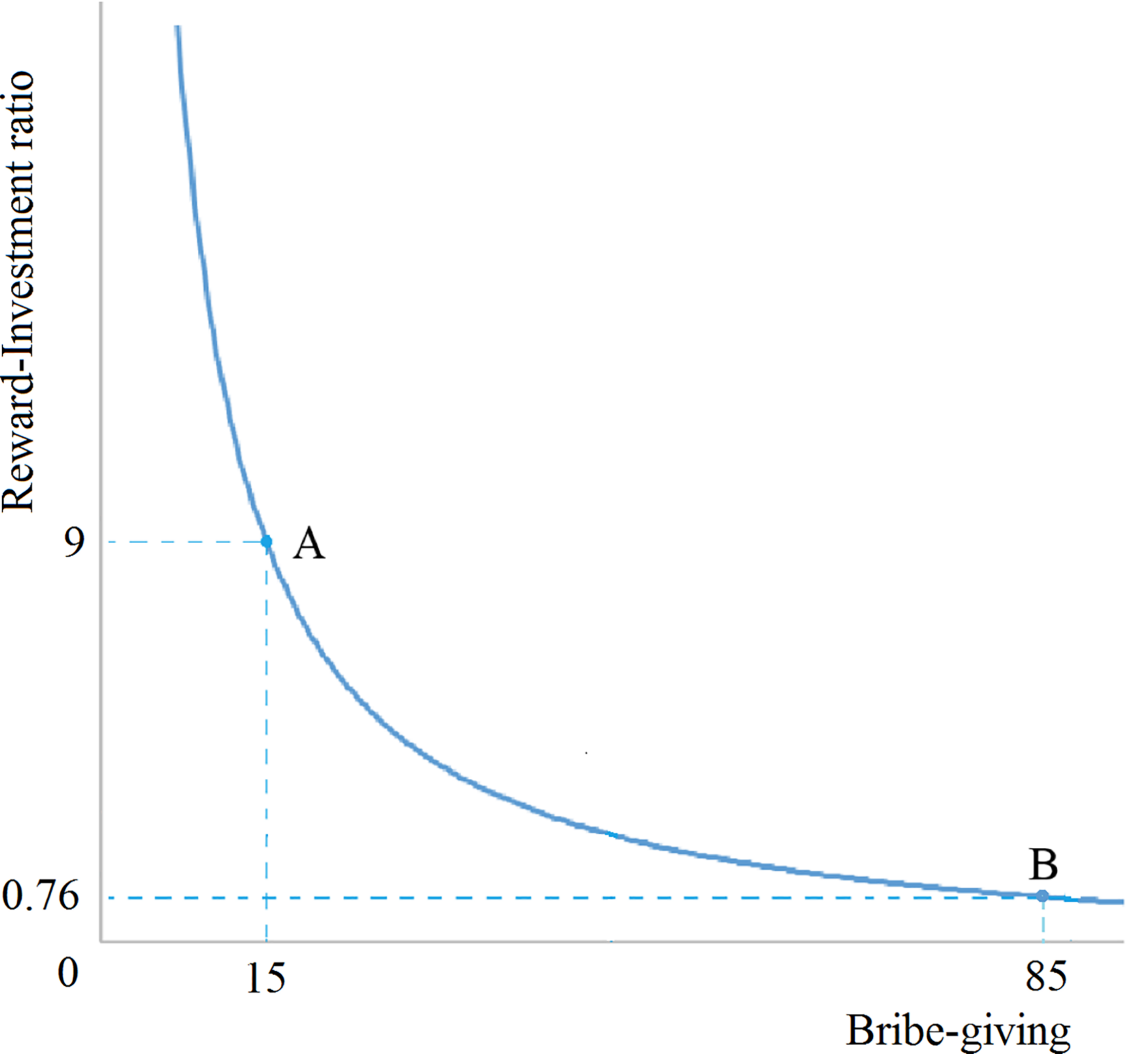
Bribe-giving utility optimization curve: RI ratio = (150—bribe-giving) / bribe-giving, represents the input-output efficiency of absolute net profit built from bribe. Point A: Low Norm Level, bribe-giving = 15 profit-ratio = 9; Point B: High Norm Level, bribe-giving = 85 profit-ratio = 0.76.

In a competitive game situation, the amount of the bribes other competitors paying is important information for participants, helping them to calculate their own bribe amount in order to optimize economic gains. We assume that individuals not only are motivated to increase their winning probability, but also want to optimize the reward-investment (RI) ratio of bribe (i.e., the absolute net profit divided by the bribe invested). In the present study, we designed the game such that while the winning probability would increase with the rise of bribe-giving, the RI ratio would shrink with the rise of bribe amount. A high RI ratio of bribe indicates winning a lot of points by giving a small bribe, whereas a low RI ratio indicates winning a few points by giving a large bribe (see the curve between profit-ratio and bribe level in Fig 1). As such, there is a decline in the marginal utility of profit that a person can derive from giving each additional unit of bribe. Under this design, it is possible that when the competitors are paying a large bribe, one may be reluctant to pay a large bribe because the RI ratio is too low. That is, one may not be willing to increase their bribe in order to win at the expense of reducing the RI ratio (see Fig 1). By contrast, when the competitors are paying small bribes, one may be more motivated to match or exceed their bribes because the RI ratio is high.

In this vein, we hypothesize that:

H1a: When the competitors are paying bribes at a high level, individuals are less likely to give a higher bribe. On the contrary, when the competitors are paying bribes at a low level, individuals are more likely to give a higher bribe.

Aside from its informational value, individuals are motivated to conform to norms because of affiliational needs, which is to say, as a tactic of impression management, individuals regulate their own behavior so that it does not deviate from others’ behavior. It is also possible that individuals conform to others’ behavior because they are motivated to be seen as in-group members. In both cases, the amount of others’ bribes serves as a valuable reference. As such, we hypothesize that:

H1b: When others are paying bribes at a high level, individuals will pay at a high level as well. When others are paying bribes at a low level, individuals will pay at a low level, too.

Although H1a and H1b are seemingly contradictory, they use different reference points. While H1a is comparing individuals’ bribe level with that of other competitors, H1b is comparing individuals’ bribe levels both before and after receiving information about the level of others’ bribes. Therefore, H1a and H1b are independent hypotheses and data may support both, neither, or only one of the two.

### Gender

There is strong evidence that men are more selfish than women based on behavioral studies [31] and meta-analysis [32]. Furthermore, women are expected to be even less selfish than they actually are [33]. Similarly, meta-analysis also found that men lied more than women in behavioral experiments [34]. That being said, gender differences in corruption and bribery remain equivocal in the literature [35] [36] [37] [38]. Swamy[35] found that when a country has a larger share of women occupying parliamentary seats, the female members of parliament upheld a higher ethical standard, which in turn resulted in a lower rate of corruption in the country. However, such gender difference was not universal. Women in Australia are less tolerant of corruption than men in Australia, but no significant gender differences were found in India, Indonesia, or Singapore. Sung [39] proposed a fair system that promotes gender equality and liberal democracy may empower women and allow them to uphold a less corrupt standard. On the contrary, Alolo [40] challenged the result that women are less corrupt; he found in Ghana that restraints in opportunities for corruption and networking make women less corrupt than men. As such, Alolo [40] attributed the gender differences observed to external constraints rather than intrinsic disposition; which is to say, to culture rather than nature. As a whole, the research is inconclusive on whether there are indeed gender differences in corruption, and if so, what exactly these underlying reasons are.

To address these inconsistent findings, the present study sought to examine whether males and females are seeking to fulfill different needs in a bribe-giving situation. It has been shown that males are more agentic (vs. communal) than females [41] [42]. Therefore, males may focus more than females on winning and outperforming others in a bribery game situation. To achieve this end, males would conceivably pay higher bribes than females. However, given the need to be accepted by others as well, males may strategically regulate their bribe-giving behaviors such that they would pay higher bribes only when the amount of their bribes is not shown to others (the private condition), but not when the amount of their bribes is shown to others (the public condition). Since females are arguably less focused than males on winning, they are plausibly less likely to increase their bribes in the private condition. Taken altogether, we hypothesize that:

H2: Males will pay higher bribes than females in the private condition but not in the public condition.

To test our hypotheses, we have conducted two studies. Study 1 tested the procedures of the bribery game and, in order to test H1a and H1b, we manipulated the bribe norm levels. In Study 2, we conducted a similar bribery game and manipulated both the norm level and bribe context (public vs. private) to test hypotheses H1a, H1b, and H2.

## Study 1

The main purpose of this study is to validate the “bribery game” and norm manipulation procedures. Specifically, we adapted the behavioral game invented by Gneezy and colleagues [17]. In addition, we created a new manipulation of the norm level—how much “bribe” the other competitors have offered (high level, low level, or no information, as control)—which allowed us to test H1a and H1b.

### Method

#### Participants

35 undergraduate students were recruited from Beijing. Mean age was 21.5 years; *SD* = 2.2. Female participants: 60%; male participants: 40%.

#### The bribery game

We adapted the game from Gneezy et al. [17], in which three players were involved, two of which were randomly chosen as players, making the third the referee. In the game, the two players have to work on a task and their performance is judged by the referee, who has absolute power to determine the winner, to whom a monetary award will be given. Therefore, the players are incentivized to win the game. In order to increase their likelihood of winning, the players are motivated to send the referee a portion of their tokens (from their endowment funds given at the beginning). Indeed, Gneezy et al. [17] found that, when the referee can keep the tokens sent by the winning player, the referee was more likely to name the player who sent more tokens as the winner. As such, this game is a suitable simulation of a bribery situation, and the number of tokens the players send can be considered a bribe.

For our new design, we modified the game to involve four participants in each session: three players and one referee. The players compete against each other in five rounds of creative tasks (see S1 Appendix), and the referee picks a winner in each round. The winner receives a prize of *p*, and the other two players receive nothing. Additionally, players may send some tokens (0 ≤ *bi* ≤ 0.6 *p*) to the referee. Importantly, the player may also choose not to send any tokens to the referee. In each round, the tokens sent from the players to the referee were counted as their bribe-giving amounts. Referees could only keep the tokens sent by the winner and had to return the other players’ bribes.

The monetary payoff of each player, *Mp*_*i*_, is described by the following formula:

$$\prod {Mp}_{i}=\left\{\begin{array}{l} -bi+p\;if\;{Mp}_{i}\;wins\\ 0\;if\;{Mp}_{i}\;loses \end{array}\right\}$$

The monetary payoff of the referee was supposed to be ∏*MR* = *b*_i*_, where *i* is the winner of each round. But, because the purpose of this study is to examine bribe-*giving*, the referee was our confederate. Also, the players were not told about the outcomes of the competition (i.e., whether they won or not) during the game, so as to avoid the feedback affecting their decisions in subsequent trials. Participants were incentivized such that the tokens they earned or kept over the five rounds would be exchanged for money at the end of the game, the exchange rate being 20 tokens for one renminbi (RMB, Chinese dollar).

In the experiment, *p* was equal to 150 tokens (RMB 7.5); the maximum number of tokens each participant could send to the “bogus” referee was therefore 90 (RMB 4.5), and the minimum was zero. To maximize the variance of bribe-giving, we used a high upper-limit bribe range. Also, to justify bribe-giving, we set a large disparity on show-up fee between players (RMB 25) and referee (RMB 5).

#### Norm-level manipulation

The participants were randomly assigned different roles, but in reality, all participants were assigned the role of “Player A.” To manipulate the norm level, participants were asked to register the tokens they sent to the referee on a form, and thus the participants saw the amounts that the “other players” allegedly sent to the referee. These amounts were bogus. In the high-level condition, the “other players” allegedly gave an average of 85 tokens to the referee (players “B” and “C” offered 80 and 90 tokens, respectively). In the low level condition, the “other players” allegedly gave an average of 15 tokens to the referee (players “B” and “C” offered 10 and 20 tokens, respectively). In the control condition, participants received no message about the other players’ bribes. If the norm level exerts conformity pressure on the participants, the participants’ bribe-giving behaviors should drift toward the norm level; that is, participants in the high-norm-level condition should give a higher bribe than those in the low-norm-level condition. However, the participants’ bribe-giving behaviors may also be affected by their calculation of economic gain.

#### Economic gain optimization

In the present experiment, sending tokens to the referee could be considered an investment for a prize of 150 tokens (the award for winning). Therefore, participants’ bribe-giving behavior should plausibly be affected by the investment-reward ratio. The reward-investment (RI) ratio of prize (*p*) and bribe (*b*) is:

RI=(p−b)b;Wherep=150tokens,

The RI ratios for the high-norm level (85 tokens) and low-norm level (15 tokens) are (as shown in Fig 1:

RI(High)=(150−85)/85=0.76

RI(Low)=(150−15)/15=9

As such, the investment-reward ratio is much greater at the low-norm level than at the high-norm level. Assuming that the three players’ productions were of similar quality, the player who gave the highest bribe would be most likely to win. Driven by economic optimization, participants should be more motivated to give a bribe higher than the norm in the low-level condition (higher than 15 tokens) than in the high-level condition (higher than 85 tokens). As such, a low vs. high norm level can influence participants’ bribe-giving behavior in different ways with regard to economic gain optimization (as espoused in H1a) and the process of conformity (as espoused in H1b).

#### Creative tasks

Participants were asked to compete on five rounds of creative tasks (see S1 Appendix) comprising generation of metaphors in Round 1 (R1), naming of abstract pictures in Rounds 2 and 5 (R2 and R5), unusual usage of objects in Round 3 (R3), and the Remote Associates Test (RAT, in Chinese) [43] in Round 4 (R4). We used creative tasks because they do not have a clear, correct answer (except RAT), which thus allows the referee to use his/her discretionary judgment to determine the winner. As a result, participants were more likely to believe that their bribes would influence the discretionary judgments of the referee. In short, creative tasks serve as a suitable means of testing bribe-giving behaviors.

#### Procedures

Each game involved four players: three “real” participants and one confederate. The participants were always assigned as “Player A” while the confederate was always named referee. Notably, the participants, confederate, and experimenter were all of the same gender in each experimental session.

At the beginning of each session, all members gathered together to learn the rules and payment procedure. Participants were told that one of them would be randomly selected as the referee, and the remaining three would be randomly assigned as players “A,” “B,” and “C.” Players would need to compete on some creative tasks in order to win a prize. The winner would be determined by the referee (our confederate) based on “the quality of the players’ answers”.

The winner received a prize of 150 tokens for the highest score in each creativity round. Each competition had a total of five rounds; each player was given 500 tokens as a “show-up fee,” or endowment fund, at the beginning, while the referee was given 100 tokens as endowment. Players were allowed to send some of their endowment tokens to the referee, but the limit in each round was 90 tokens. The referee would keep the tokens sent by the winner of each round, while the tokens sent from the losing players would be returned to them. The results of all the rounds would not be released until the end of the entire session, at which point the participants were given monetary payments based on the tokens they had won (or kept) over the course of all five rounds. The participants’ monetary payments were calculated using the following formulas:

- For players: Endowment (500 tokens) + (150 tokens × number of wins)—tokens given to the referee in the winning rounds.
- For referee: Endowment (100 tokens) + tokens from winners of 5 rounds.

The exchange rate was 20 tokens for each renminbi. Note that the referee was always our confederate. Referees learned the rules together with the other participants at the beginning. After learning the rules, the participants were tested about their knowledge of the rules. After passing the quiz satisfactorily, the participants played the game independently in separate booths.

In each round, players first worked on the creative task for three minutes. Then they put their answer sheet into an envelope together with the number of tokens (0 to 90) they decided to send to the referee. The experimenter came to collect the envelope and asked the players to write down the number of tokens they had sent to the referee on a registration form. To manipulate the norm level, the participants were shown how much the “other players” had given to the referee, starting after the second round (these numbers were bogus). As a manipulation check, at the end of the whole session, the participants were asked to recall how much on average the other players have given to the referee.

At the end of the game, the participants were debriefed and given a final payment which was equal to: Endowment (500 tokens) + (150 × 2 winning times)—the two highest numbers of tokens sent to the referee.

### Results and discussion

#### Descriptive statistics

Beginning after the second round, players were given bogus feedback on the number of tokens sent by the other players from the registration form. Therefore, the participants’ bribe-giving behaviors in Round 1 (R1) and Round 2 (R2) could be considered a baseline, whereas those in Round 3 through Round 5 (R3, R4, and R5) could be influenced by the norm manipulation. Therefore, behavioral change was tested by comparing the means from R3 through R5 with those from R1 and R2. Significant difference in number of tokens sent between R3 and R5 as opposed to R1 and R2 was expected if the social norm level influences bribe-giving behavior. Table 1 shows the means and standard deviations among the three manipulation groups.

**Table 1:** Mean difference of bribe-giving under three conditions.

	N	Round1 Mean (SD)	Round 2 Mean (SD)	Round 3 Mean (SD)	Round 4 Mean (SD)	Round 5 Mean (SD)
High	11	43.64(22.0)	43.64(22.5)	67.27(20.0)	62.73(23.7)	71.82(20.4)
Low	12	48.33(35.4)	44.17(37.0)	36.67(26.1)	37.5(29.3)	45.83(30.6)
Control	12	44.17(26.4)	44.17(26.4)	42.50(29.9)	47.5(37.7)	54.17(22.7)
*F*	—	.10	.00	4.50*	1.92	3.18^#^
*eta*^*2*^	—	.01	.00	.22	.11	.17

*: *p* < .05.

^#^: *p* < .08.

#### Hypothesis testing

**Fig 2:**
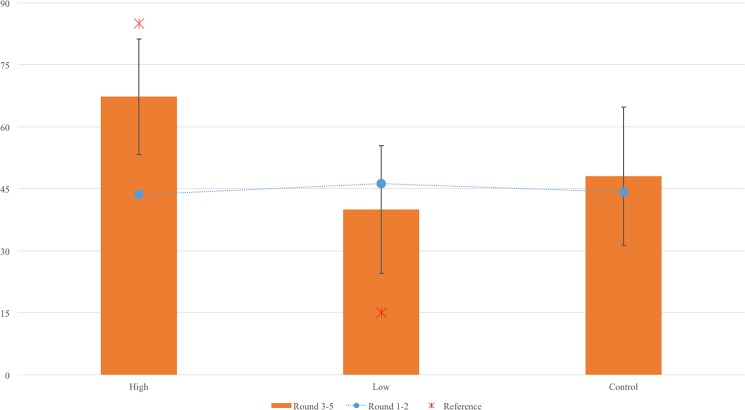
Means of token-sent under 3 conditions comparison of Round 3–5 with Rounds 1 and 2.

To test H1a, we examined whether participants would pay a lower bribe in R3-R5 than the norm in the high-norm-level condition, and whether they would pay a higher bribe in R3-R5 than the norm in the low-norm level. As shown in Fig 2, as predicted, the confidence interval of participants’ bribes was significantly lower than the reference point of 85 in the high-norm condition. By contrast, it was significantly higher than the reference point of 15 in the low-norm condition. As such, H1a was supported.

To test H1b, a 2 (R1-R2 vs. R3-R5) × 3 (norm level) × 2 (gender) repeated measure ANOVA was performed on the bribe amounts. The interaction effect of round × norm level was significant (*F* = 8.39, *p* = .00, *eta*^*2*^ = .38). Follow-up analysis showed that participants increased their mean bribe level in the high-norm condition significantly from before receiving the norm information vs. after (*F* = 40.11, *p* = .00, *eta*^*2*^ = .80), whereas the mean bribe level in the low-norm condition did not drop significantly (*F* = 1.11, *p* = .32, *eta*^*2*^ = .10, ns.). This pattern of findings partially supports H1b. It is possible that because participants were willing to pay a higher bribe when others paid a lower bribe (akin to H1a), thereby countering the conformity effect, and resulting in a non-significant drop in bribe level in the low-norm condition.

Interestingly, overall, the average bribe amount for the control condition laid between those of the high- and low-norm conditions, and did not change much between R1-R2 and R3-R5. This gives credibility to our norm manipulation.

We have also counted the number of times participants gave zero bribe (i.e., not sending any tokens to the referee). The frequency was low (10.29% on average). Participants in the low norm level condition showed the highest frequency of giving zero bribe (16.67%), compared to 1.82% in the high norm level, and 11.67% in the control condition.

In short, the bribery game and the manipulation procedures seem to support most of our predicted results. However, because the manipulation of norm level was performed only after Round 2, the test of bribe level change may have been limited. Therefore, we decided to move the manipulation forward one round in Study 2. In addition, because we did not manipulate context in this study, the gender effects were not significant (e.g., round × gender: *F* = .08, ns.). For Study 2, we manipulated the public vs. private context to test the gender differences hypothesized in H2.

## Study 2

To test all our hypotheses, we manipulated both the bribe level (high vs. low) and context (public vs. private). Furthermore, we sought to examine if participants’ performance on the creative tasks would affect their bribe-giving behaviors as well. We’ll elaborate our argument next.

### Performance

In the bribery game, participants compete at a creative task in each round. The participants are told that the referee will select the winner based on his or her evaluations of the quality of the participants’ answers. As such, it is possible that participants would give a larger bribe in order to remedy a low-quality answer. If this is the case, we should observe a *negative* correlation between the amount of bribes paid and the quality of the answers, which can be measured in terms of the participants’ confidence in their answers and also the objective quality of the answers. On the other hand, it is also possible that participants would give a larger bribe in order to ensure that their high-quality answer would not be undermined by others’ bribes. If this is the case, we should observe a *positive* correlation between bribes amounts and quality of answers. To test these two opposing ideas, we adopted a latent growth curve model to test how participants changed their bribe-giving behavior across the five rounds of competition as a function of their performance on the creative task in each round, together with the norm level, norm context manipulation, and participant gender.

### Method

#### Participants

133 undergraduate students from Beijing were recruited as participants. Mean age was 19.35 years; *SD* = 1.90. Female participants: 53.4%; male participants: 46.6%.

#### The bribery game and manipulations

We used a bribery game similar to that in Study 1, in which three actual participants and one confederate were asked to participate in each experimental session. Again, the gender of the participants, confederate, and experimenter was matched each time. We modified the procedures in manipulating the norm level and norm context (private vs. public).

For the *norm-level manipulation*, we included only the high- and low-norm-level conditions and the manipulation was the same as in Study 1. However, the bogus norm information was given after each round from Round 1 (R1). Also, as a manipulation check, participants were asked to estimate the number of tokens sent by their opponents (the other two players) after each round. Because the participants were informed of their opponents’ bribes amount after R1, significant differences between high- and low-norm-level conditions were found in R2 (*F* = 61.79, *p* < .01, *eta*^*2*^ = .32), R3 (*F* = 133.54, *p* < .01, *eta*^*2*^ = .51), R4 (*F* = 123.19, *p* < .01, *eta*^*2*^ = .49) and R5 (*F* = 140.56, *p* < .01, *eta*^*2*^ = .52) but not in R1 (*F* = .00, *ns*.). Significant positive correlation was found between participants’ estimation of opponents’ bribe amounts and their own bribe amounts in R1 through R5, in both high-level and low-level conditions (range of *r* = [.56, .60], *p* < .01), providing preliminary support to the idea that perceived social norm would affect individuals’ behavior.

To manipulate the norm context (private vs. public), all participants were assigned the role of “Player C.” As such, while the participants could see the (bogus) amounts that Players A and B sent to the referee, they expected that the amount they registered would not be viewed by the other players in the private condition because a new registration form was used for each round; conversely, they believed their amount would be viewed by the other players in the following rounds in the public condition because the same form was used for all rounds. As a manipulation check, at the end of the game, we asked participants that, “Do you think other competitors would know the amount of token you have sent?” 87% participants in the public condition reported “yes”, whereas 70% participants in the private condition reported “no”. These results suggest that the norm context manipulation was by and large effective.

In sum, this experiment used a 2 norm level (high vs. low) × 2 norm context (private vs. public) between-subjects design. Participants were randomly assigned to one of the four groups.

#### Supplementary surveys

To check if the participants in the four manipulation conditions held similar perceptions and beliefs about the game, participants were asked to fill out the following survey questions after the game:

*Belief in bribe utility*. To examine whether participants in the four conditions believed to a similar extent that giving bribes would help them win, participants were asked: “To what extent do you believe that sending more tokens to the referee will increase your chance of winning the game?” Score from 1 = “absolutely not” to 6 = “absolutely yes.” ANOVA testing showed that there was no significant difference in this belief across the four manipulation conditions (*F* = .63, *p* = .60). Participants in general believed in a relatively high bribe utility in the four conditions (*M* = 4.57, *SD* = 1.71). Interestingly, male participants held a marginally stronger belief in bribe utility than female participants (*F* = 3.79, *p* = .05). Also, there was a significant correlation between belief in bribe utility and amount of bribe sent by the participants to the referee (*r* = .20, *p* < .05).

*Belief of tokens’ importance*. To examine if participants in the four conditions believed to a similar extent that the referee would be biased by the amount of the bribe sent, participants were asked: “To what extent do you believe that the referee’s judgment is based on the number of tokens sent?” Score from 1 = “absolutely not” to 6 = “absolutely yes.” Again, ANOVA testing showed that there was no significant difference in this belief across the four manipulation conditions (*F* = .59, *p* = .62). Participants in general believed that the referee’s judgments were based on the amount of the bribes sent in the four conditions (*M* = 5.15, *SD* = 1.36).

*Belief in answer-quality importance*. To examine if participants in the four conditions believed to a similar extent that the referee’s judgment was based on the quality of their answers, participants were asked: “To what extent do you believe that referee’s judgment is based on the quality of answers in the creative task?” Score from 1 = “absolutely not” to 6 = “absolutely yes.” There was a significant difference in this belief across the four manipulation conditions (*F* = 4.12, *p* = .01, *eta*^*2*^ = .08). Participants in the high-norm level believed more strongly that the referee’s judgments were based on their answer quality than did those in the low-norm level (*F* = 6.83, *p* = .01, *eta*^*2*^ = .50). In the public-norm context, participants believed in answer quality importance marginally more than in the private context (*F* = 3.85, *p* = .05, *eta*^*2*^ = .03). Interesting, male participants held consistently less belief in answer quality than did female participants.

#### Creative tasks

We used the same creative tasks as in Study 1. In addition, to assess their subjective confidence about their own performance, the participants were asked after each round, “To what extent do you believe that you have produced a more creative answer than other opponents?”

To assess participants’ actual performance on the creative task, the participants’ answers in R1, R2, R3, and R5 were evaluated independently by two independent raters in three domains: originality, novelty and practicality. The raters were asked to rate independently from 0 = “not at all” to 7 = “very much.” Originality describes how unique and imaginative the answers are; novelty represents the rareness of the answers; practicality represents the applicability and reasonability of the answers. Creativity score was the mean value of the three domains. Inter-rater reliability (Kendall’s correlations) between raters ranged from .50 to .71 in originality, from .52 to .76 in novelty, and from .55 to .60 in practicality. The internal reliability (Cronbach’s alpha) for those three domains was .87, .91 and .91, respectively. The ratings from the two raters were averaged to create a score for each participant in each round. For the Remote Associates Test (RAT) in R4, raters compared participants’ answers with objective answers to score their quality. The higher the score, the more creative the participant’s actual performance.

### Results and discussion

#### Descriptive statistics on bribe-giving change

At the beginning of the experiment, R1 behavior represents the baseline of bribe-giving, where the tokens sent to the referee were not influenced by social norms. From R2 to R5, participants were given the norm-level and norm-context manipulations. The descriptive behavior change was tested by comparing the means from R2 to R5 with the baseline at R1. Table 2 showed the means and standard deviations across the four manipulation conditions through the five rounds.

**Table 2:** Mean difference in bribe-giving under four conditions.

	N	Round1	Round 2	Round 3	Round 4	Round 5
		Mean (SD)	Mean (SD)	Mean (SD)	Mean (SD)	Mean (SD)
High–Private	35	41.71	56.29	55.43	57.43	54
		(-26.4)	(-28.5)	(-29.73)	(-31.28)	(-32.56)
High–Public	27	32.59	49.26	57.04	45.19	57.41
		(-25.21)	(-30.88)	(-32.2)	(-36.41)	(-35.69)
Low–Private	40	42	38.75	36	32.5	39.5
		(-29.11)	(-25.74)	(-26.87)	(-27.53)	(-27.64)
Low–Public	31	38.71	30	32.58	31.29	30
		(-32.53)	(-20.17)	(-26.95)	(-23.06)	(-22.06)
*F*	—	0.71	6.26**	6.32**	5.97**	5.74**
*eta*^*2*^	—	0.02	0.13	0.13	0.12	0.12

*: *p* < .05.

**: *p* < .01.

#### Testing hypotheses 1a and 1b

**Fig 3:**
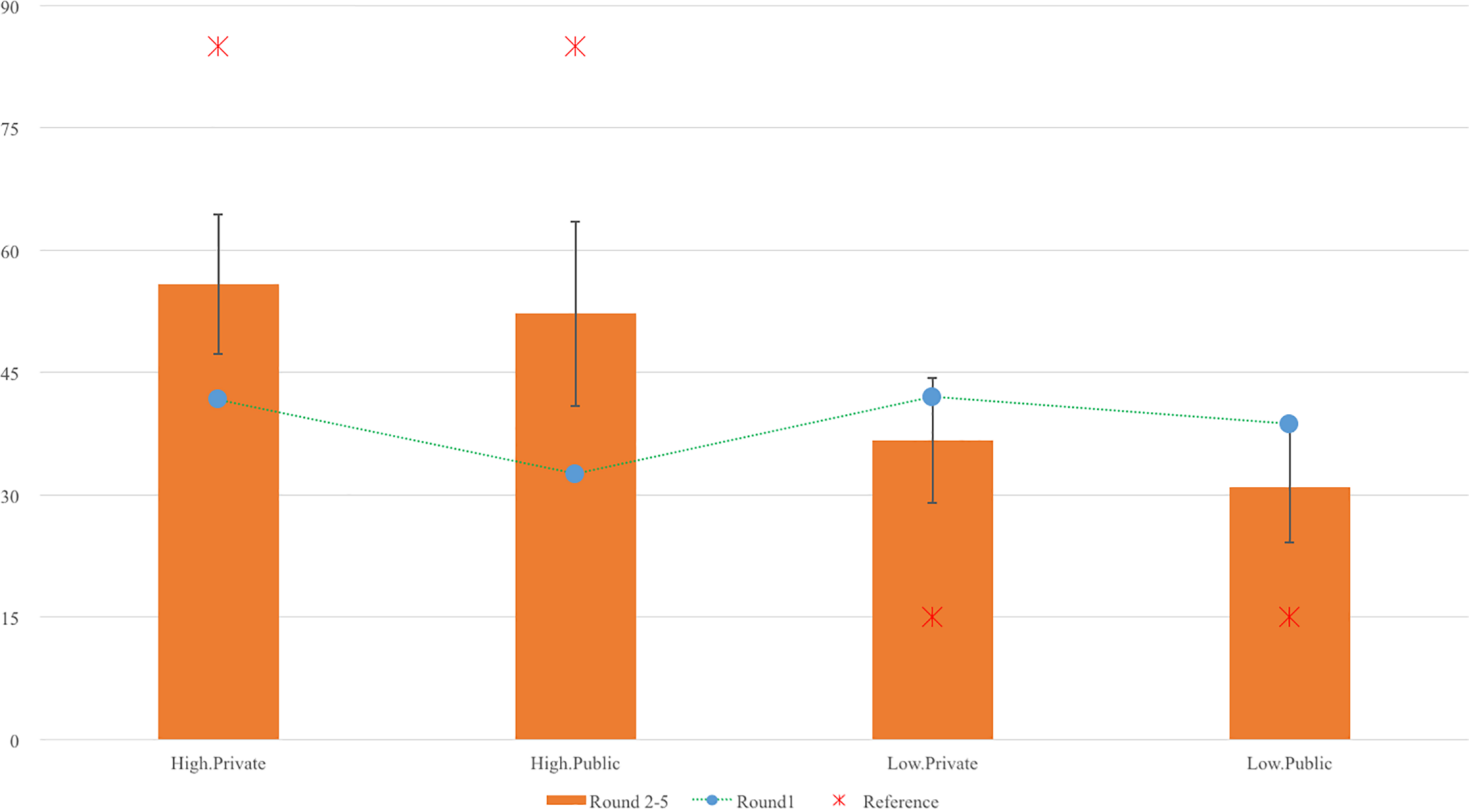
Means of token-sent under 4 conditions comparison of Round 2 through Round 5 with Round 1.

To test H1a, we examined R2-R5 to ascertain whether participants would pay a below-norm bribe in the high-norm-level condition (both public and private contexts) and an above-norm bribe in the low-norm-level condition (both public and private contexts). As shown in Fig 2, indeed, as predicted, the 95% confidence interval of participants’ bribes was significantly lower than the reference point of 85 in the high-norm condition (see Fig 3). By contrast, it was significantly higher than the reference point of 15 in the low-norm condition. As such, H1a was supported.

To test H1b, a 2 (R1 vs. R2-R5) × 2 (norm level) × 2 (norm context) × 2 (gender) ANOVA was conducted on the bribe amount. First, the interaction effect of round × norm level was significant (*F* = 20.62, *p* = .00, *eta*^*2*^ = .14). The norm level influences the change in token-sent behavior compared with the baseline. Follow-up analysis showed that participants increased their mean bribe level in the high-norm condition significantly from before receiving the norm information versus after (*F* = 23.42, *p* = .00, *eta*^*2*^ = .27). Likewise, participants decreased their mean bribe level in the low-norm condition, although the change was only marginally significant (*F* = 3.08, *p* = .08, *eta*^*2*^ = .04). Moderate increases were reported in High level under Private (Cohen's d = .55) and Public (.73) conditions, while small decline were reported in Low level under Private (Cohen's d = .20) and Public (.29) conditions. This result by and large supports H1b. As in Study 1, it is possible that participants’ willingness to pay a higher bribe when others paid a low one (akin to H1a)—thereby countering the conformity effect—resulted in a marginal drop in bribe level in the low-norm condition.

We have also counted the number of times participants gave zero bribe (i.e., not sending any tokens to the referee). The frequency was low (9.92%). Importantly, the distribution of the zero bribe was not systematically associated with the norm level, norm context, or gender (chi-sq = .74, df = 3, p = .86).

In short, the pattern of findings was highly similar to those revealed in Study 1. Again, H1a and H1b were supported in general. Participants were affected by the norm information and adjusted their bribe amount accordingly. They were driven both by their economic gain optimization and conformity tendency. On the one hand, they adjusted their bribe amount in the direction of the norm level (increased in the high-norm condition and decreased in the low-norm condition). However, considering the marginal return, participants were more likely to pay a higher-than-the-norm bribe in the low-norm condition than in the high-norm condition.

#### Testing hypothesis 2

**Fig 4:**
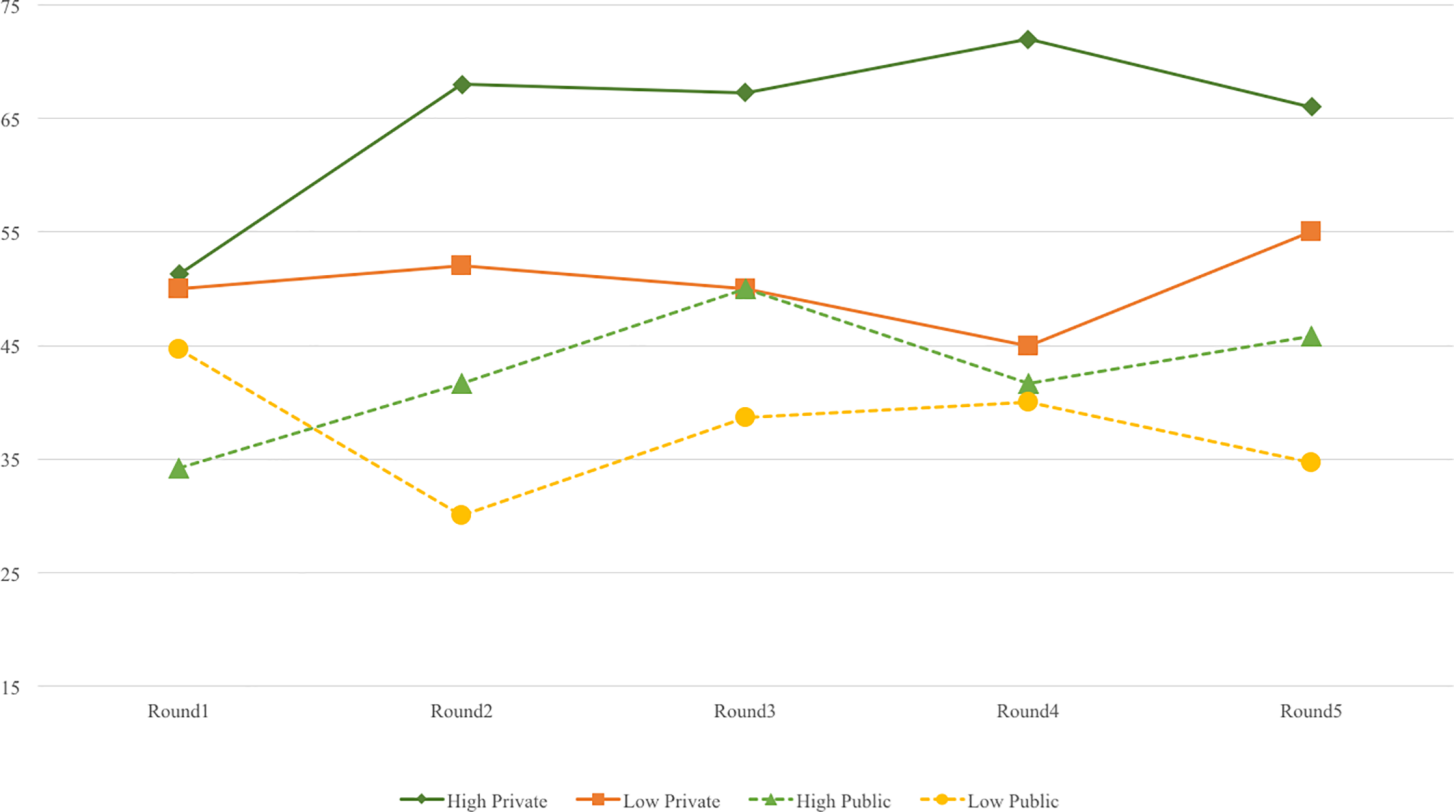
Token-sent in 5 rounds under 4 conditions for male participants.

**Fig 5:**
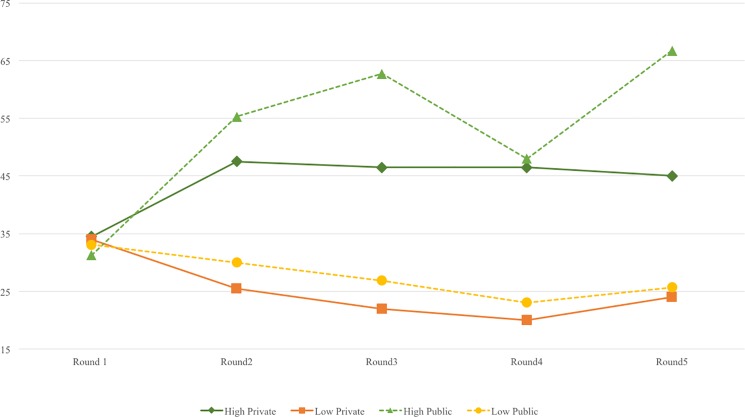
Token-sent in 5 rounds under 4 conditions for female participants.

From the 2 (R1 vs. R2-R5) × 2 (norm level) × 2 (norm context) × 2 (gender) ANOVA conducted on the bribe amount, the gender main effect was significant (*F* = 10.02, *p* = .00, *eta*^*2*^ = .07), such that females in general gave a significantly lower bribe (*M* = 37.75, *SD* = 24.29) than males (*M* = 50.16, *SD* = 26.41). This main effect was further qualified by a significant gender × norm context interaction effect (*F* = 5.09, *p* = .03, *eta*^*2*^ = .04). The gender × norm level interaction was insignificant (*F* = 1.54, *p* = .22, *eta*^*2*^ = .01, ns). The means of tokens-sent in five rounds under the four manipulation conditions for males and females are shown in Fig 4 and Fig 5. As predicted in H2, males paid significantly higher bribes in R2-R5 (*M* = 58.14, *SD* = 24.23) than females (*M* = 34.63, *SD* = 22.68) in the private condition (*F =* 16.32, *p* = .00, *eta*^*2*^ = .18). But, in the public condition, there was no significant gender difference (for females, *M* = 41.77, *SD* = 26.04; for males, and *M* = 39.81, *SD* = 25.93. *F =* .27, *p* = .61, *eta*^*2*^ = .01, ns.) This pattern supports H2.

#### Trajectory change of behavior and creative performance

Fig 4 and Fig 5 show the bribe level changes of the 2 (norm level) × 2 (norm context) × 2 (gender) groups across the five rounds. To examine the impacts of manipulation and task performance on participants’ bribe-giving behavior, we used a latent growth curve model (LGCM) of Mplus 7.0 to understand changes in trajectory over the five rounds.

The LGCM treats the tokens sent (TS) across five rounds as a series of bribe-giving behavioral outcomes determined by latent growth variables. The changes in bribe-giving behavior are described by initial value, change rate, and change direction, which are estimated by Quadratic LGCM in terms of intercept, slope, and quadratic index, respectively. In the model, the latent intercept variable (I) representing the initial TS was estimated by setting indicator paths observed from the first round to the last to be “1”. A latent slope variable (S) representing linear change in TS was estimated by setting indicator paths from the observed first through fifth rounds to be 0, 1, 2, 3, and 4, respectively. A latent quadratic variable (Q) representing quadratic change in TS was estimated by setting indicator paths from the observed first through fifth rounds to be 0, 1, 4, 9, and free estimate, respectively. By setting the path from the initial TS (I) to the S and Q at “0”, the “I” represents the TS level at the onset of the study. [44]. Although the sample size of this study is not abundant, Bayesian method is used for its capable of providing less-biased estimates with small samples [45].

**Fig 6:**
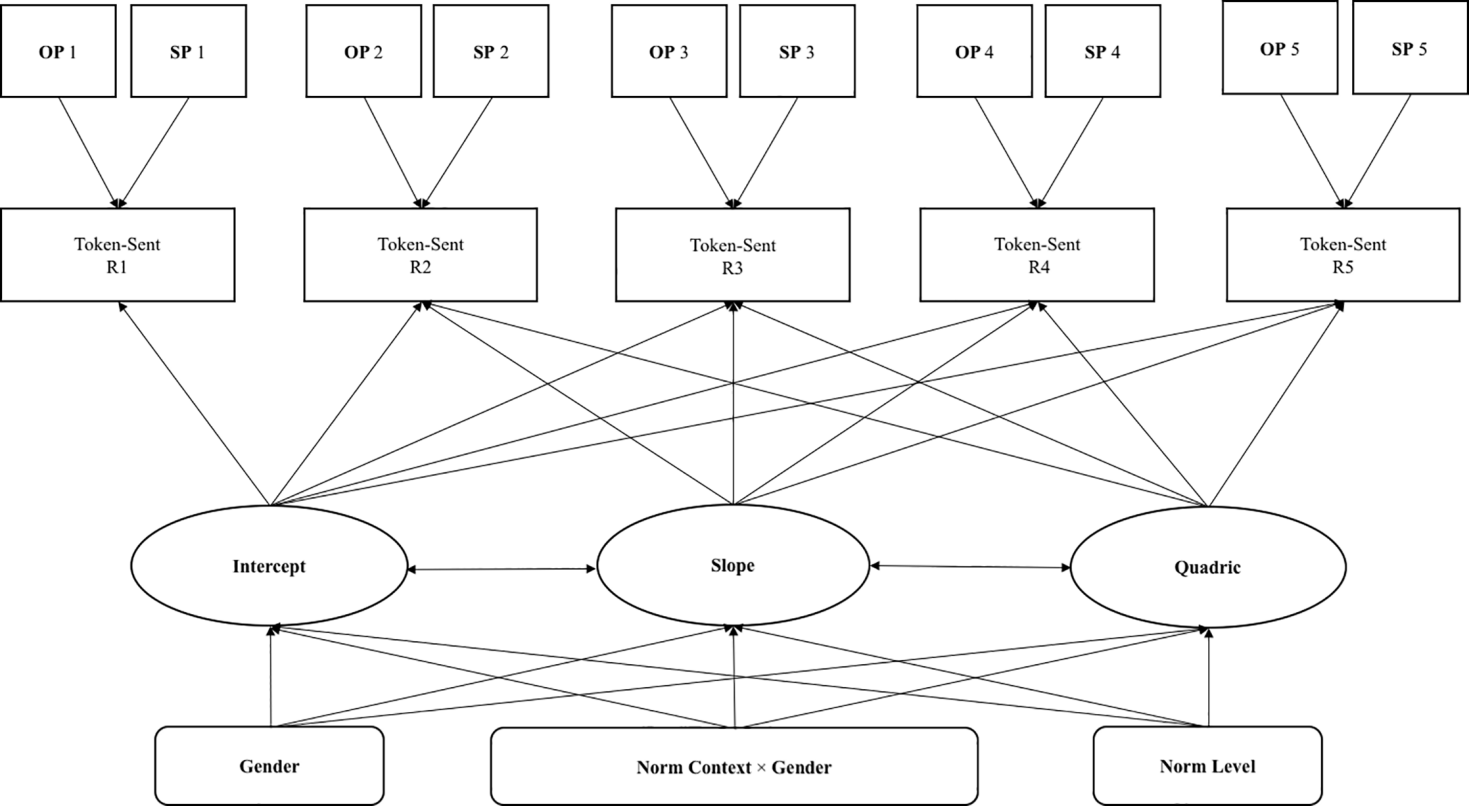
Hypothetical quadratic latent growth-curve model with covariates.

The norm level, gender, and gender × norm contexts were included as time-invariant variables, because they continued influencing across the five rounds. We also included time-varying variables in the model, including subjective confidence (SP) about creative performance and objective evaluation on creative performance (OP) in each round. The hypothetical path diagram for our LGCM model is shown in Fig *6*.

**Fig 7:**
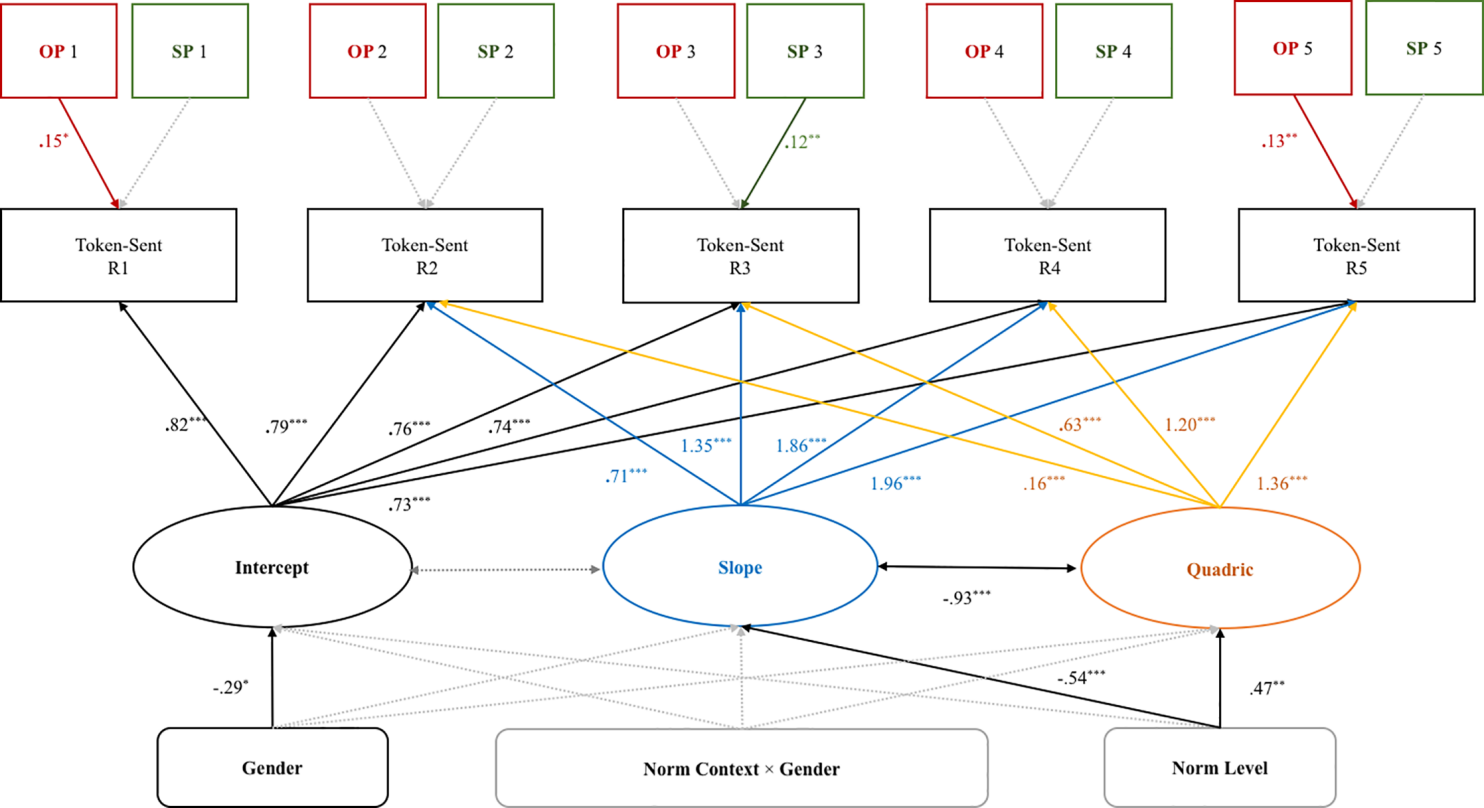
Latent growth curve model with significant standardized path coefficients.

The estimation of model results is shown in Fig 7. The 95% confidence interval for the difference between the observed and the replicated chi-square values is [-17.38, 47.21]. The posterior predictive *p*-value is 0.173, showing that the data can well fit the model we proposed.

In the LGCM, the standardized estimated mean value of intercept is 1.67, *SE* = .54, *p* = .00, latent slope = 2.30, *SE* = .71, *p* = .00, latent quadratic = -2.29, *SE* = .96, *p* = .00. The latent slope negative correlated with latent quadratic, indicating the steep behavioral change also has a slow rate of change.

The significant intercept variable showed that the TS baseline was significantly predicted by gender. Male participants gave higher bribes than females at the beginning of each experiment. Social norm level significantly predicted the slope and quadratic of change. Participants in the low-norm level decreased their bribe amount at a higher rate of change throughout the five rounds, while participants in the high-norm level increased their bribe amount at a lower rate of change.

In terms of the performance, the results showed that participants with superior objective performance paid significantly higher bribes in the first and final rounds (R1 and R5), while subjective confidence in performance significantly predicted a higher bribe in the middle round (R3).

In sum, the LGCM results provide more information to understand bribe-giving behavior; these changes were determined by latent growth variables. First, gender predicted the dispositional difference (intercept) in the entire competition process. Males offered higher bribes than females from the beginning. Unexpectedly, the interaction effect of gender × norm context did not predict the change in bribe amount across the five rounds. Second, norm level predicted linear and quadratic change in bribe amount from R2 through R5. Participants in the low-norm level decreased their bribe-giving at a faster rate than did those in the high-norm level. Moreover, high subjective confidence and better actual creative performance were associated with higher bribes sent in each round, suggesting that the bribes were seen as insurance rather than remedy.

## General discussion

This research sought to gain a deeper understanding of the mechanism underlying bribery; specifically, we examined the influences of social norm and gender on bribe-giving behavior in an incentivized game simulating a bribery situation. We reasoned that social norm provides information for strategic planning and for impression management, and that these two influences would drive different patterns of bribe-giving behaviors. For strategic planning, we predicted that people would try to outperform other competitors when the cost is relatively low, but not when the cost is high. By contrast, for impression management, we predicted people would follow others’ bribe-giving level. To test these ideas, we manipulated whether other competitors paid bribes at a high-versus-low level in two studies. Findings consistently revealed that participants gave a significantly less-than-norm bribe (< 85 tokens) in the high-norm condition, but gave a significantly higher-than-norm bribe (> 15 tokens) in the low-norm condition, suggesting that people were motivated to outperform other competitors when the marginal cost was low. In addition, participants increased their mean bribe level significantly after they received the norm information in the high-norm condition, but decreased their mean bribe level in the low-norm condition, although this drop was not statistically significant. This may be because participants were willing to pay higher bribes than others at the low-norm level, thereby countering the conformity effect and resulting in a non-significant drop in their bribes in the low-norm condition. Taken as a whole, both studies provide consistent evidence that social norms affect bribe-giving behaviors via their informational (strategic planning) and affiliational (impression management) influences.

In the current research, we also examined whether males and females were differentially affected by social norms. We reasoned that males, being more agentic than females, would defy the norm in order to win when the amount of their bribe was kept in private, but would conform to the norm when it was made public. To test these ideas, we manipulated the bribe-giving context (private vs. public). As predicted, findings revealed that males gave larger bribes in the private context than in the public, whereas females gave smaller bribes in both private and public contexts. The latent growth curve model also revealed that gender, norm level, and participants’ creative performance influenced the trajectory change of bribe-giving behaviors across the five rounds of competition, such that male (vs. female), high (vs. low) level, and better (vs. worse) performance were linked to an increasing trajectory of bribes across the five rounds.

### Implications and future research

Findings from the current research have several implications for the notion that bribery is a cultural phenomenon. Relatedly, our findings provide supportive evidence that people do conform to others’ level of bribe-giving, supporting Abbink [30], who posited that social-norm conformity could predict bribe-offering behavior. That is, a high bribery rate breeds more bribery. Even for nations that have low bribery levels to begin with, people would probably still increase their bribe in order to win, if there were no regulations prohibiting bribery. Moreover, Gelfand and colleagues [46] found that people in tight cultures were more likely to adhere to social norms than people in loose cultures. Therefore, a high bribery rate would breed more bribery in tight cultures than in loose cultures. Likewise, it is also possible that a low bribery rate would be better maintained in tight cultures than in loose cultures. Future research can examine these ideas.

Focus theory differentiates between two types of social norms: descriptive and injunctive [23]. Descriptive norms refer to the prevalence of certain behaviors in a group and can be reflected through statistical frequencies. For instance, divorce can be represented as a descriptive norm, i.e., how statistically prevalent it is in a country. By contrast, injunctive norms refer to the social acceptance of certain behaviors in a group, and are often linked with moral judgments. For instance, divorce may be seen as unacceptable and morally incorrect in some cultures. In the present research, we have only manipulated the descriptive norms of bribery; we have not manipulated the injunctive norms surrounding bribery. It would be interesting to examine whether setting up an injunctive norm of “zero-tolerance” toward bribery is an effective way to curb bribe-giving behavior. According to our findings, lowering the descriptive norm of bribe level did not eliminate bribe-giving because people are still willing to pay at a higher-than-norm level in order to win. Instead, it is possible that injunctive norms, together with harsh penalties, would better eradicate bribery. This possibility merits future investigation because recent research [47] has shown that injunctive norms are much stronger than descriptive norms, at least in the case of the so-called Trade-Off Game, in which people have to decide between two opposite norms: being equitable or being efficient.

In many societies, bribe-giving is treated as an “implicit rule” rather than an illegal activity; examples are abundant, such as giving “red pockets” to doctors in China and tipping officials in driver’s license examinations in India. The prevalence of bribe-giving may also reflect pluralistic ignorance; that is, although people in a given culture may disapprove of the bribery norm individually/in private, they may believe that other people approve of bribery because they have observed it to be common in their society [48] [49]. As such, bribery can be entrenched in the social system and can harm a society in ways that go beyond the acts of bribery themselves. Take the Ming Dynasty in China as an example. Under this government, bribery was seen as an act of showing loyalty to the group. If an official refused to be involved in bribery, he was seen as deviant from his peers, and was thus excluded. Therefore, the government was left with corrupt officials [50].

Our findings also shed light on how to understand the inconsistent gender differences found from previous bribery studies [35] [38]. For example, in a meta-analysis, Capraro [34] found that males tend to be more dishonest than females in a variety of deception situations, independently of the consequences of lying. Stensota and colleagues [51], however, posit that gender difference is more pronounced in the legislative arenas of greater ambiguity. Likewise, we found that males gave significantly higher bribes than females when the context was private. These patterns may be driven by males’ greater focus on agency (i.e., power and achievement), which is often shaped through socialization in many societies. As a result, males may be more motivated to bribe given that the bribe will enhance achievement and the context allows (in private, with little social or legal penalty).

This research also addresses the issues of relation between individual performance and bribe-giving behavior. On the one hand, bribe-giving could be seen as remedy to inferior performance and lack of confidence. For example, many school buildings collapsed during the 2008 Sichuan earthquake, killing hundreds of children. Subsequently, it was discovered that the contractors of those buildings gave bribes to officials to avoid meeting building safety standards [15]. That is, bribes were used to compensate for inferior performance. On the other hand, bribe-giving could be seen as reinforcement of superior performance and high confidence. For instance, Wal-Mart has allegedly of paid bribes in developing countries (Mexico, China, and others) to expand its business. *The New York Times* called Wal-Mart “an aggressive and creative corrupter” [52]. In our study, we found that participants tended to use bribes as a form of insurance to help them win rather than as a remedy for low performance.

However, an alternative explanation to this finding could be that more creative participants are more likely to commit unethical behavior (e.g., giving bribes). According to Gino and colleagues [53] [54], creative and dishonest behaviors both involve breaking rules. They indeed found that highly creative individuals were more likely to engage in dishonest behaviors than were their less creative counterparts. It is possible that creative individuals may be better able to find ways to break rules, and also be more tolerant of rule-breaking behaviors, including bribery. Consistently, we also found that participants’ creativity level (as indicated by their objective creative performance in the game) was positively correlated with their bribe amount. This alternative explanation is worthy of further investigation.

### Limitations and conclusion

The main limitation of the present experiment is a lack of ecological validity in the laboratory. In particular, participants may not interpret giving tokens in the game to be exactly the same as giving bribes, and thus may not consider the act to be violating ethical codes. That being said, however, some participants did indeed voice their moral concerns about giving tokens, especially in the public condition. These individuals may have viewed the bribe-giving through an ethical frame (whether it violates fairness) rather than a business frame (whether it is just a business deal). To increase the ecological validity, it may be useful to manipulate injunctive norms (i.e., by issuing penalties) in future research. These factors were out of the scope of the current research.

To conclude, the present research examined how situational factors and personal factors may influence bribe-giving behaviors. Specifically, we examined how social norms (e.g., the amount of bribes others gave) and gender affected individuals’ bribe-giving behavior in a behavioral game. Findings from two studies consistently revealed that individuals conformed to the norm level of bribe-giving (high vs. low), while maintaining a relative advantage for economic benefits. Also, males gave larger bribes in the private context than in the public, whereas females gave smaller bribes in both private and public contexts. Taken together, these findings suggest that social norms and social context do indeed have significant influence on bribe-giving behaviors.

## Supporting information

S1 Appendix Creativity task. (PDF)

S1 Dataset Minimal data set. (XLSX)
