# Supplementary material for: Norm, gender, and bribe-giving: Insights from a behavioral game
Source: PLoS One. 2017 Dec 22;12(12):e0189995. doi: 10.1371/journal.pone.0189995 (PMC5741253; doi:10.1371/journal.pone.0189995)
Supplement: S1 Appendix — (PDF) [file pone.0189995.s001.pdf]

Appendix

Creative Tasks

Round 1 (R1): Generation of Metaphors

Often used in writing and communication, analogy is the relating of two things from different domains through a similarity. For example: Friendship is similar to rock. Relation: friendship is as strong and indestructible as rock.

Please find two analogies for "time" and list their relations.

Please make your answers as original, unique, and reasonable possible. The referee will score your answer from 0-100 according to those standards.

(a) Time is similar to _________________

The relation is: _______________________________________

(b) Time is similar to _________________

The relation is: _______________________________________

Round 2 (R1): Naming of Abstract Pictures

Here is a mysterious graffito. It may need your imagination to guess what it is. Someone has already given this graffito a name; your task is to give it another five names.

Please make your answers as original, unique, and reasonable as possible. The referee will score your answer from 0-100 according to those standards.

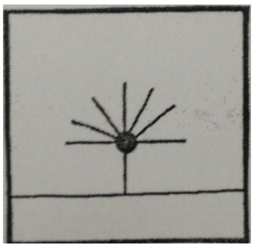

Someone named it "a queer flower."

Please list up to five new names:

1.____
2.____
3.____
4.____
5.____

Round 3 (R3): Usage of Objects

The important qualifications of creativity are originality and practicality. Please list some original and practical uses of the daily objects below.

Please make your answers as original, unique, and reasonable as possible. The referee will score your answer from 0-100 according to those standards.

1. Plastic bag:

____

2. Chair:

____

Round 4 (R4): Remote Associates Test

Creative thinking usually involves associational factor restructuring. For example, the characters “疗,” “防,” and “统” can be combined with another common character, “治,” to make Chinese words, including “治疗,” “防治,” and “统治.”

Please find the common character that can be combined with the three given Chinese characters to make up frequently used words. Note that the words cannot be personal names, place names, quantifiers, or proper nouns.

Please make your answers as original, unique, and reasonable as possible. The referee will score your answer from 0-100 according to those standards.

1. 害，青，伐

____

2. 立，言，调

____

Round 5 (R5): Naming of Abstract Pictures

Here is a mysterious graffito. It may need your imagination to guess what it is. Someone has already given this graffito a name; your task is to give it another five names.

Please make your answers as original, unique, and reasonable as possible. The referee will score your answer from 0-100 according to those standards.

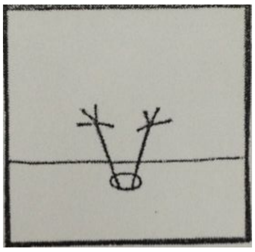

Someone named it “trapped upside-down bird.”

Please list up to five new names:

1.____

2.____

3.____

4.____

5.____
